# Supplementary figures and images for: Role of oxidation of excitation-contraction coupling machinery in age-dependent loss of muscle function in Caenorhabditis elegans
Source: eLife. 2022 May 4;11:e75529. doi: 10.7554/eLife.75529 (PMC9113742; doi:10.7554/eLife.75529)

## Full uncut gels for Figure 1

*E*

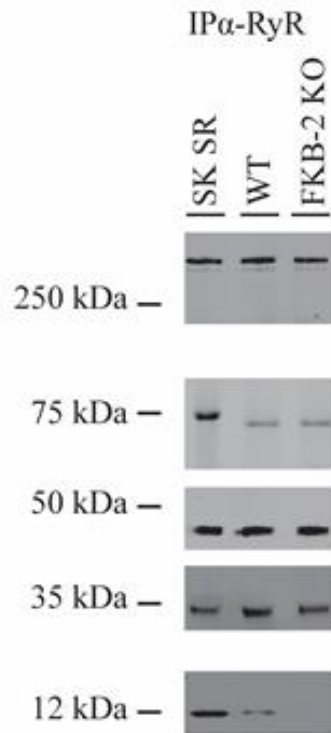

*F*

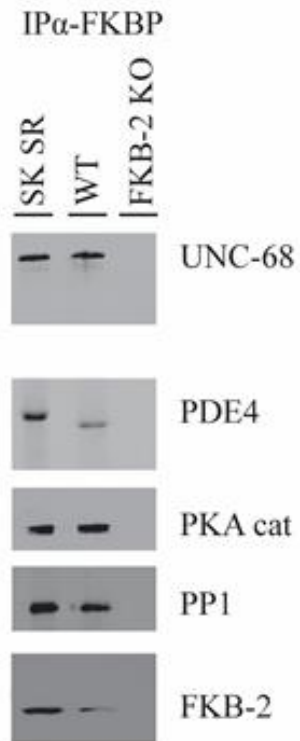

IP $\alpha$ -RyR

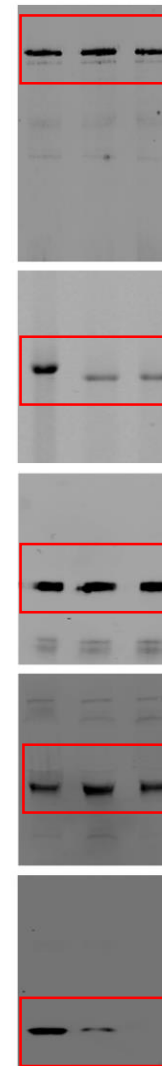

IP $\alpha$ -FKBP

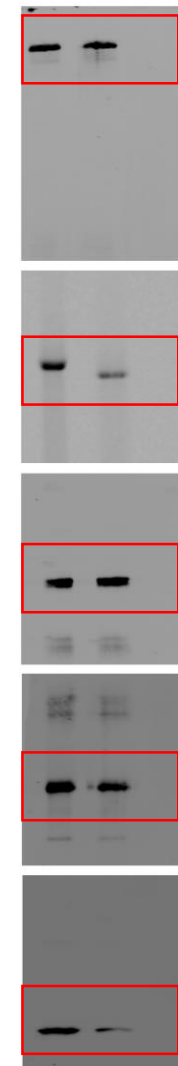

UNC-68

UNC-68

PDE4

PDE4

PKA cat

PKA cat

PP1

PP1

FKB-2

FKB-2

Supplement: Figure 1—source data 1. [file elife-75529-fig1-data1.pdf]

Full uncropped gel for figure 2

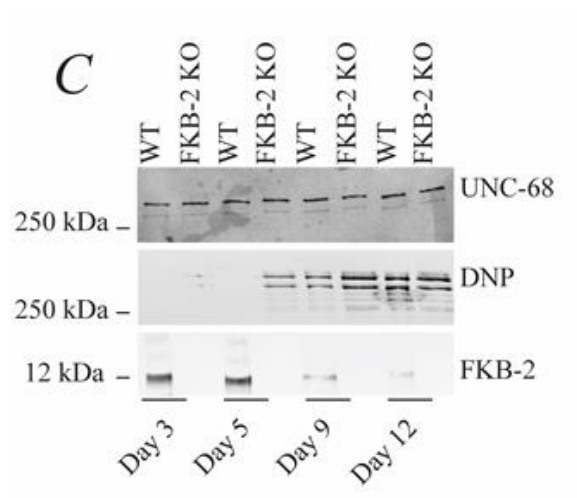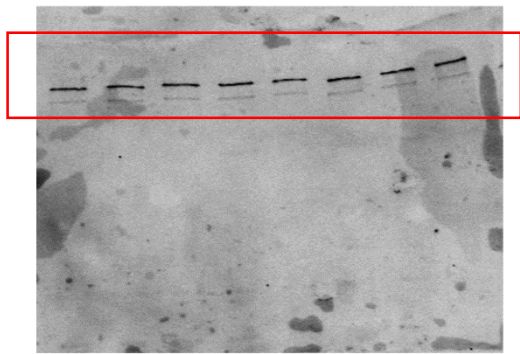

UNC-68

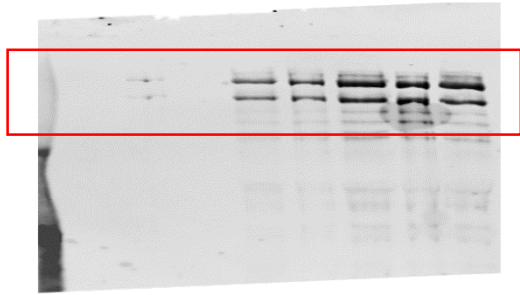

DNP

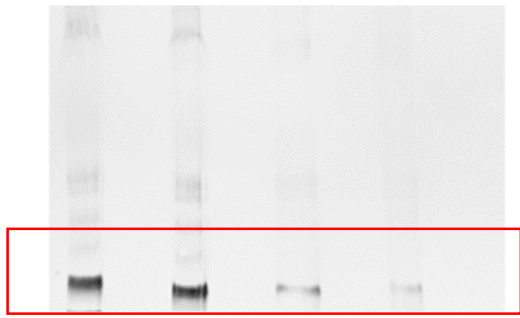

FKB-2

Supplement: Figure 2—source data 1. [file elife-75529-fig2-data1.pdf]

## Full uncut gels for Figure 3

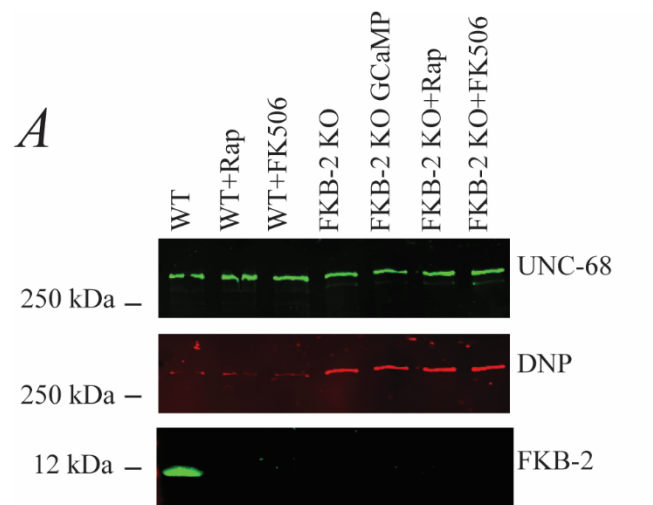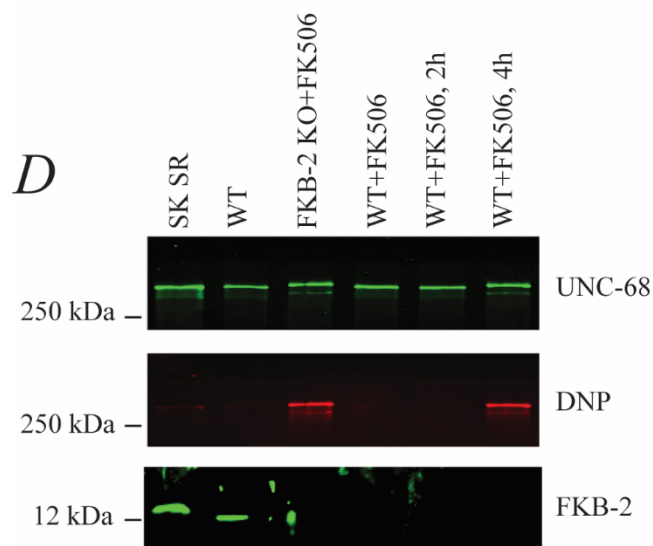

Figure 3A

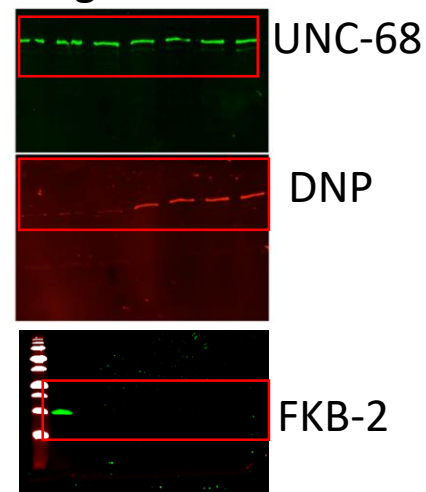

Figure 3D

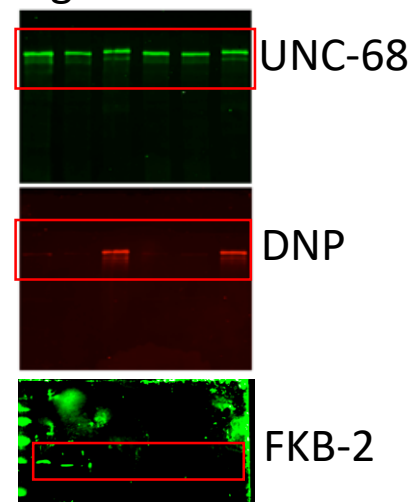

Supplement: Figure 3—source data 1. [file elife-75529-fig3-data1.pdf]

Full uncropped gel for figure 4

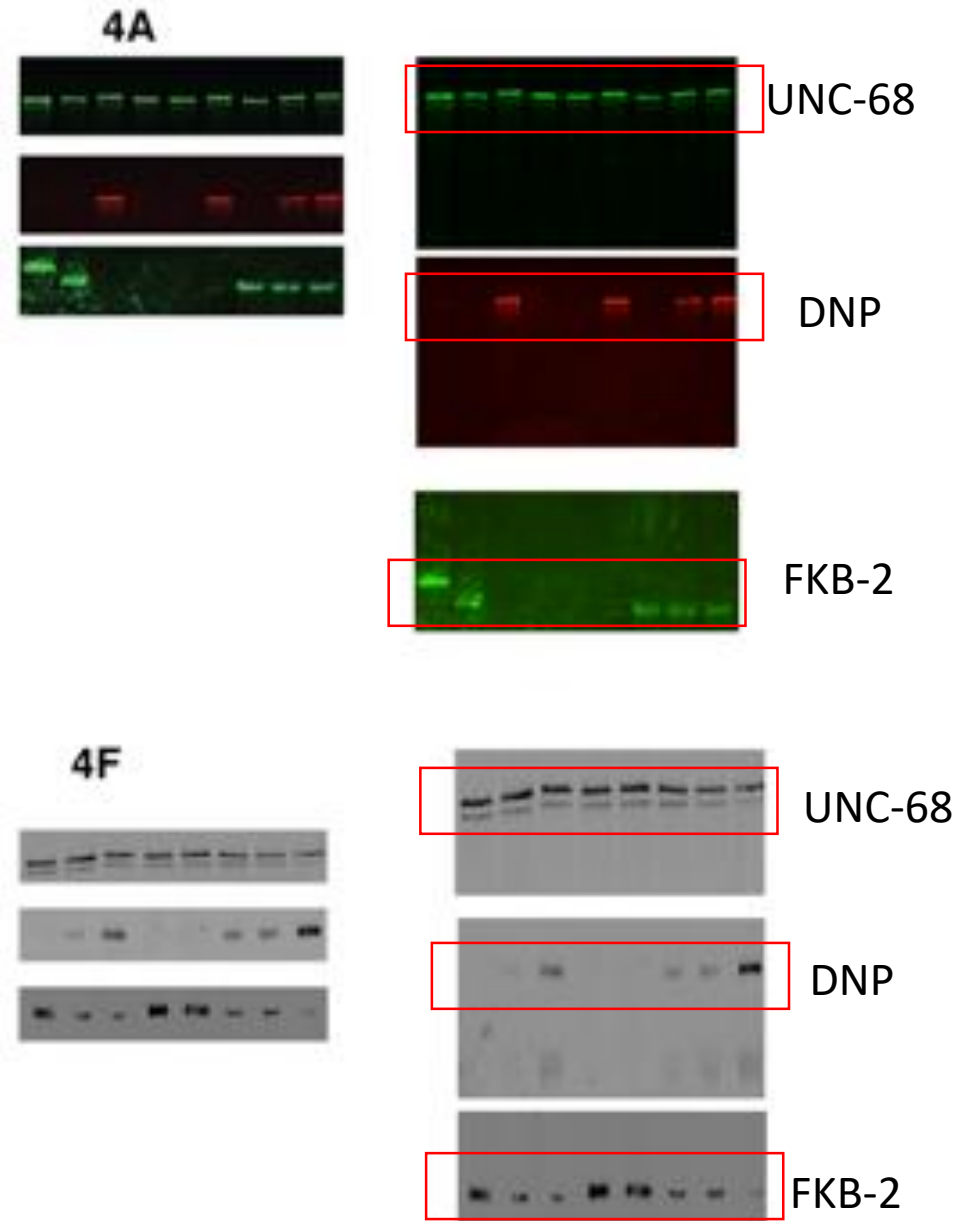

Supplement: Figure 4—source data 1. [file elife-75529-fig4-data1.pdf]

# Full uncropped gel for figure 5

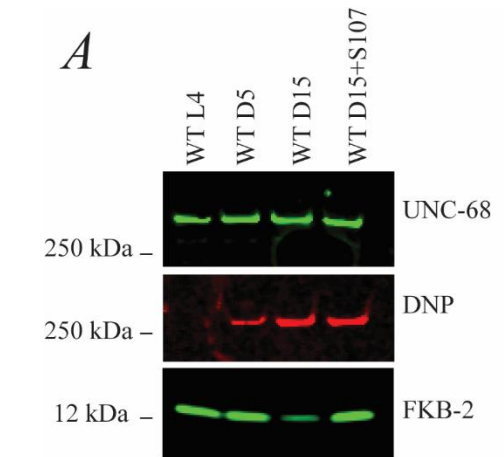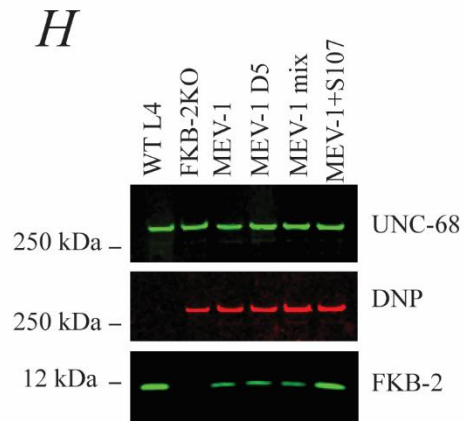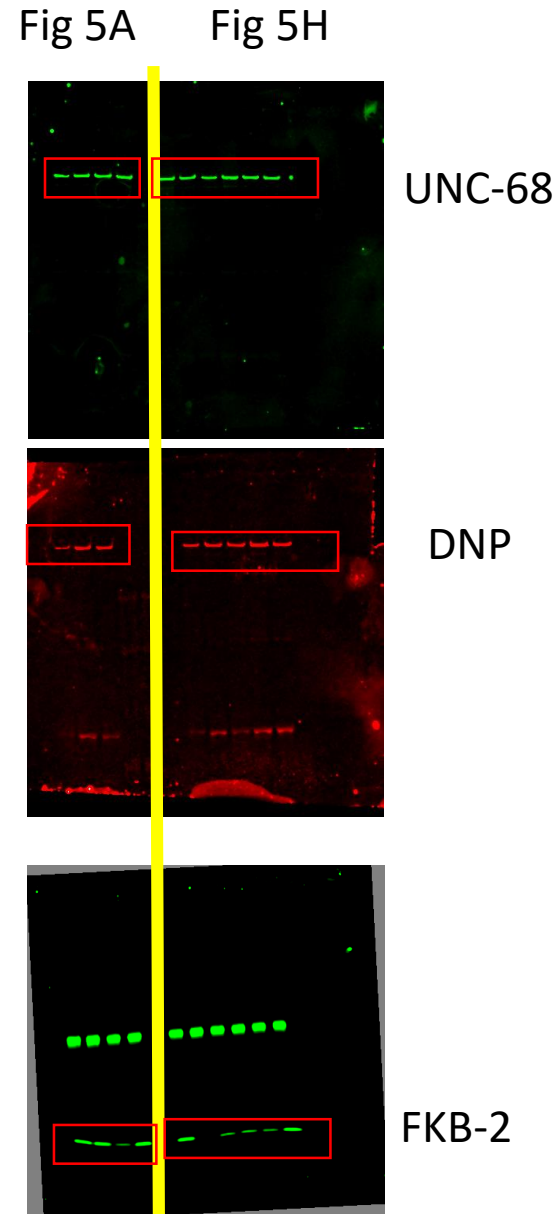

Supplement: Figure 5—source data 1. [file elife-75529-fig5-data1.pdf]
