# Supplementary material for: Role of oxidation of excitation-contraction coupling machinery in age-dependent loss of muscle function in Caenorhabditis elegans
Source: eLife. 2022 May 4;11:e75529. doi: 10.7554/eLife.75529 (PMC9113742; doi:10.7554/eLife.75529)
Supplement: Supplementary file 1. — Comparison of homologous oxidative and antioxidative genes between C. elegans, mouse and human. Criteria of comparison includes the function, the subcellular location, the enzymatic activity, mutation induces disrupted phenotype and percentage of homology. [file elife-75529-supp1.docx]

***Supplementary File 1*:**

| **Protein** | ***FOXO (Forkhead Box Class O)*** | ***CAT (Catalase)*** | ***SOD (Superoxide Dismutase)*** | ***PRDX/PRX (Peroxiredoxin)*** | ***TRX(Thioredoxin)*** | ***GLRX (Glutaredoxins)*** |
| --- | --- | --- | --- | --- | --- | --- |
| **Function** | Transcription Factor | Antioxidative Enzyme | Antioxidative Enzyme | Antioxidative Enzyme | Antioxidative Enzyme | Antioxidative Enzyme |
| **Human** | FOXO1,3,4,6 | CAT | SOD1, 2, 3 | PRDX/PRX 1,2,3,4,5,6 | TRX | GLRX 1,2,3,5 |
| **Murine** | FOXO1,3,4,6 | cat | Sod 1, 2,3 | Prdx | Trx | Glrx 1,2,3,5 |
| ***C. elegans*** | DAF-16 | Ctl 1, 2,3 | Sod 1,2,3,4,5 | prdx 2, 3, 6 | trx 1,2,3,4,5 | glrx 3, 5, 10, 21, 22 |
| **Location human vs *C. elegans*** | Nucleus  Vs  Nucleus | Peroxisome vs Peroxisome | Cytoplasm, nucleus and mitochondria vs cytoplasm and mitochondria | Cytoplasm vs cytoplasm | Nucleus and cytoplasm vs nucleus | Cytoplasm, mitochondria vs mitochondrial matrix |
| **Enzymatic activity** | None | Human: ~50U/mg (skin) and 98.6 MU/l (blood); Mouse: 4-18U/mg (Liver); Worm: 35-55U/mg over lifespan | SOD-1 Human: ~30 U/mg (skin)  Mouse: 4 U/mg; SOD-2 | Human & Rat: 40 (mmol/min/mg) | Human: 0.15/100ug x 10^3 | Human:10nmol/min/mg in lung tissues  Mouse : Identical to human |
| **Disruption**  **phenotype in**  ***C. elegans*** | Mouse: Homozygote has diverse defects; secondary infertility, decreased glucose uptake, mild anemia. Worm: impaired dauer formation. | Ctl2 KO causes progeria, decreased lifespan | Overexpression induces resistance to oxidative stress and increased lifespan | Prdx-2 KO Worm: sensitive to oxidation. decreased lifespan. | Trx ko worms: reduced lifespan, sensitive to oxidative stress (paraquat) | Not tested |
| **Homology** | 22.14% ID, (to FOXO3) | 60.53 ID to clt1 | 55.35 to SOD1 | 72.86 ID to prdx 6 | 33.91% ID to trx1 | 45.10% ID to glrx3 |
| **References** | ([93](#_ENREF_93), [94](#_ENREF_94)) | ([95](#_ENREF_95)) | ([96](#_ENREF_96)) | ([97](#_ENREF_97), [98](#_ENREF_98)) | ([99-101](#_ENREF_99)) | ([102](#_ENREF_102)) |

| **Protein** | ***Glutathione S-transferase*** | ***NAD-dependent protein deacetylase (SIR)*** | ***Dual specificity mitogen-activated protein kinase kinase*** | **Nuclear respiratory factor 1**  **(NRF1)** | ***Dual oxidase 1*** | **D-beta-hydroxybutyrate dehydrogenase** |
| --- | --- | --- | --- | --- | --- | --- |
| **Function** | Antioxidative Enzyme | reduction of the 'Lys-16' acetylation of histone H4 | Kinase activity involved in oxidative stress | mitochondrial DNA transcription and replication | N/A | Oxidative Enzyme |
| **Human** | GSTA 1-5/  GSTM 1-5 | SIRT 1-7 | MAP2K1 | NRF1 | Oxidative Enzyme | BDH1 |
| **Murine** | GSTA 1-7/  Gstm 1-4 | Sir 1-7 |  | Nfr1 | DUOX1 | Bdh1 |
| ***C. elegans*** | Gst 1-44 | Sir 2.1-2.4 | Mek 1, 2 | Skn 1 | Duox1 | N/A |
| **Location human vs**  ***C. elegans*** | Cytosol vs Cytoplasm and mitochondria | Nucleus, mitochondria and cytoplasm vs  Nucleus and cytoplasm | Nucleus and mitochondria vs Cytoplasm | cytoplasm vs  Nucleus and mitochondria | bli-3 | Mitochondria |
| **Enzymatic activity** | 121 nmol/min/mg in human  N/A for C-elegans | N/A | N/A | N/A | Plasma membrane vs plasma membrane | N/A |
| **Disruption**  **phenotype in**  ***C. elegans*** | RNAi-mediated knockdown causes an increase in Mn^2+^-mediated dopaminergic CEP neuron degeneration | Reduces the longevity  RNAi-mediated depletion results in an increase of 'Lys-16' acetylation of histone H4 (H4K16ac) | defects in egg laying infertility  Reduced lifespan with stress.  No obvious phenotype in absence of stress | RNAi-mediated knockdown causes an increase in Mn^2+^-mediated dopaminergic CEP neuron degeneration and a reduction in expression levels of glutathione S-transferase gst-1 | RNAi-mediated knockdown + proline ROS production, reduces the expression of skn-1 and reduces longevity | N/A |
| **Homology** | 29.28% ID to GSTA1 | 28.17% ID to SIR1 |  | 12.20% ID to NRF1 | 32.78% | N/A |
| **Refs** | ([103](#_ENREF_103), [104](#_ENREF_104)) | ([105-107](#_ENREF_105)) |  | ([104](#_ENREF_104), [108](#_ENREF_108), [109](#_ENREF_109)) | ([110](#_ENREF_110), [111](#_ENREF_111)) |  |

| **Protein** | **Xanthine Oxidase** | **NO Synthase** | **Cytochrome P450** | **Hydroxyacid oxidase 1** | **SDH (Succinate Dehydrogenase)** |  |
| --- | --- | --- | --- | --- | --- | --- |
| **Function** | Oxidative Enzyme | Oxidative Enzyme | Oxidative Enzyme | Oxidative Enzyme | Oxidative Enzyme |  |
| **Human** | XDH | NOS 1,2,3 | CYP (Multiple) | HAO1 | SDHA, SDHB, SDHC, SDHD |  |
| **Murine** | Xdh | Nos 1,2,3 | Cyp (multiple) | Hao1 | Sdha, Sdhb, Sdhc, Sdhd |  |
| ***C. elegans*** | xdh-1 | Unknown | cyp-(23-37) | Unknown | sdha-1, sdhb-1, sdhd-1 |  |
| **Location human vs**  ***C. elegans*** | Peroxisome and Extracellular region vs Cytosol |  | ER vs Unknown |  | Mitochondria VS Mitochondria |  |
| **Enzymatic activity** | Rat: 4.4 ccm oxy uptake/unit time/unit weight of intestine | Human: .5-2.5 pmol/min/mg (brain) Mouse: Normalized to 1 | Multiple | --- | 1.45 ferricyanide reduced/min.mg wetwt. (Euthyroid) |  |
| **Disruption**  **phenotype in**  ***C. elegans*** | Unavailable |  | Unavailable |  | Unavailable |  |
| **Homology** | 46.54% |  | 28.97% |  | 69.12% |  |
| **Refs** | ([112](#_ENREF_112)) |  | ([113-116](#_ENREF_113)) |  | ([117](#_ENREF_117)) |  |
