# Supplementary material for: Role of oxidation of excitation-contraction coupling machinery in age-dependent loss of muscle function in Caenorhabditis elegans
Source: eLife. 2022 May 4;11:e75529. doi: 10.7554/eLife.75529 (PMC9113742; doi:10.7554/eLife.75529)
Supplement: Supplementary file 2. — Comparison of amino acid abundance in the C. elegans UNC-68 and the human RyR1 calcium channels. Number and percentage of Serines and methionines for each species are shown in red. [file elife-75529-supp2.docx]

***Supplementary File 2***

| **UNC-68 (*C. elegans*)** |  |  | **RyR1 (Human)** |  |
| --- | --- | --- | --- | --- |
| **Number of amino acids** | 5187 |  | 5038 |  |
| **Molecular weight** | 589102.13 |  | 565175.53 |  |
| **Amino acid composition:** | Number | % | Number | % |
| Ala (A) | 333 | 6.4 | 384 | 7.6 |
| Arg (R) | 265 | 5.1 | 294 | 5.8 |
| Asn (N) | 216 | 4.2 | 170 | 3.4 |
| Asp (D) | 302 | 5.8 | 253 | 5 |
| Cys (C) | 90 | 1.7 | 100 | 2 |
| Gln (Q) | 235 | 4.5 | 205 | 4.1 |
| Glu (E) | 417 | 8 | 476 | 9.4 |
| Gly (G) | 305 | 5.9 | 363 | 7.2 |
| His (H) | 133 | 2.6 | 133 | 2.6 |
| Ile (I) | 281 | 5.4 | 203 | 4 |
| Leu (L) | 533 | 10.3 | 558 | 11.1 |
| Lys (K) | 314 | 6.1 | 228 | 4.5 |
| **Met (M)** | **179** | **3.5** | **145** | **2.9** |
| Phe (F) | 251 | 4.8 | 207 | 4.1 |
| Pro (P) | 194 | 3.7 | 271 | 5.4 |
| **Ser (S)** | **376** | **7.2** | **296** | **5.9** |
| Thr (T) | 256 | 4.9 | 231 | 4.6 |
| Trp (W) | 61 | 1.2 | 63 | 1.3 |
| Tyr (Y) | 155 | 3 | 141 | 2.8 |
| Val (V) | 291 | 5.6 | 317 | 6.3 |
| Pyl (O) | 0 | 0 | 0 | 0 |
| Sec (U) | 0 | 0 | 0 | 0 |
